# Loss of Consciousness and Righting Reflex Following Traumatic Brain Injury: Predictors of Post-Injury Symptom Development (A Narrative Review)

**DOI:** 10.3390/brainsci13050750

**Published:** 2023-04-30

**Authors:** Rina Berman, Haley Spencer, Martin Boese, Sharon Kim, Kennett Radford, Kwang Choi

**Affiliations:** 1 Center for the Study of Traumatic Stress, Uniformed Services University, Bethesda, MD 20814, USA; rina.berman.ctr@usuhs.edu; 2 Program in Neuroscience, Uniformed Services University, Bethesda, MD 20814, USA; haley.spencer.ctr@usuhs.edu; 3 Daniel K. Inouye Graduate School of Nursing, Uniformed Services University, Bethesda, MD 20814, USA; martin.boese@usuhs.edu (M.B.); kennett.radford@usuhs.edu (K.R.); 4 F. E. Hébert School of Medicine, Uniformed Services University, Bethesda, MD 20814, USA; sharon.kim@usuhs.edu; 5 Department of Psychiatry, Uniformed Services University, Bethesda, MD 20814, USA

**Keywords:** mild traumatic brain injury, righting reflex, loss of consciousness, injury severity, prognosis

## Abstract

Identifying predictors for individuals vulnerable to the adverse effects of traumatic brain injury (TBI) remains an ongoing research pursuit. This is especially important for patients with mild TBI (mTBI), whose condition is often overlooked. TBI severity in humans is determined by several criteria, including the duration of loss of consciousness (LOC): LOC < 30 min for mTBI and LOC > 30 min for moderate-to-severe TBI. However, in experimental TBI models, there is no standard guideline for assessing the severity of TBI. One commonly used metric is the loss of righting reflex (LRR), a rodent analogue of LOC. However, LRR is highly variable across studies and rodents, making strict numeric cutoffs difficult to define. Instead, LRR may best be used as predictor of symptom development and severity. This review summarizes the current knowledge on the associations between LOC and outcomes after mTBI in humans and between LRR and outcomes after experimental TBI in rodents. In clinical literature, LOC following mTBI is associated with various adverse outcome measures, such as cognitive and memory deficits; psychiatric disorders; physical symptoms; and brain abnormalities associated with the aforementioned impairments. In preclinical studies, longer LRR following TBI is associated with greater motor and sensorimotor impairments; cognitive and memory impairments; peripheral and neuropathology; and physiologic abnormalities. Because of the similarities in associations, LRR in experimental TBI models may serve as a useful proxy for LOC to contribute to the ongoing development of evidence-based personalized treatment strategies for patients sustaining head trauma. Analysis of highly symptomatic rodents may shed light on the biological underpinnings of symptom development after rodent TBI, which may translate to therapeutic targets for mTBI in humans.

## 1. Introduction

Traumatic brain injury (TBI) is a condition caused by a blow or jolt to the head as a result of motor vehicle accidents, falls, blast injuries, or other mechanisms, and is a leading cause of death and disability in the United States [1]. TBI may be classified as mild, moderate, or severe based on the duration of loss of consciousness (LOC), alteration of consciousness (AOC), and post-traumatic amnesia (PTA); structural imaging; and the Glasgow Coma Scale (GCS), which assesses depth of consciousness on a scale of 3–15 whereby 13–15 indicates mild, 9–12 moderate, and 5–8 severe TBI [2]. The vast majority of TBI cases (80–90%) are considered mild; however, the perceived mildness of the condition produces barriers for patients to receive proper treatment [3]. Although mTBI-related symptoms can resolve without intervention, a subset of patients develops persistent symptoms, which is referred to as post-concussive or post-concussion syndrome (PCS) [4,5]. Approximately 10–40% of mTBI cases are accompanied by LOC lasting less than 30 min [6,7], and LOC may be associated with adverse outcomes. Thus, the current review will discuss clinical studies of the association between the presence and duration of LOC and adverse outcomes in mTBI patients.

The righting reflex is the innate tendency of an animal placed on its back to flip over and return to its feet. In preclinical literature, the loss of righting reflex (LRR) duration is commonly used as a surrogate for LOC in humans and a proxy measure of TBI severity [8]. Rodent TBI literature, unlike human mTBI literature, does not have classification criteria for TBI severity based on LRR. A review of rodent and human TBI studies suggested guidelines for LRR corresponding to each TBI severity; however, studies cited in this review had wide variations in LRR and were inconsistent in how they defined TBI severity [8]. Additionally, experimental factors, such as models with drastically different injury profiles [8,9], make strict LRR cutoffs for classification of TBI impractical. Furthermore, LRR is a highly heterogeneous measure subject to large individual variability, such that two rodents experiencing the same injury with identical parameters may have drastically different LRR. Because LRR is a measure of injury severity, it stands to reason that rats with high LRR would generally have worse symptoms. As such, the preclinical literature review section will examine studies in which LRR was associated with TBI outcome measures in rodents.

## 2. Literature Search Strategy

The current narrative review is a representative overview of both clinical mTBI and preclinical TBI. Clinical studies were identified using search terms “mild traumatic brain injury” AND “loss of consciousness” in PubMed and Google Scholar. The aim of this portion of the review was to compare mTBI patients with and without LOC. Studies examining multiple injury severities (i.e., mild moderate, and severe) were permitted as long as some analysis for mTBI patients with and without LOC was conducted. Preclinical studies were found using the search terms “righting reflex” AND “traumatic brain injury” AND “predictor”; “righting reflex” AND “traumatic brain injury” AND “behavior” AND “predictor”; “righting reflex” AND “traumatic brain injury” AND “correlational analysis” AND “behavior”; and “righting reflex” AND “traumatic brain injury” AND “correlation” in PubMed and Google Scholar. Only studies using rodents (i.e., mice and rats) were included. All searches were conducted without the restriction of time period.

## 3. Loss of Consciousness in Clinical mTBI Studies

The following studies compare mTBI with LOC to mTBI without LOC and/or control groups to determine the relationship between LOC and outcome variables after mTBI. The studies contained in this section are summarized in Table 1. 

### 3.1. Cognitive and Memory Deficits

The presence and duration of LOC are associated with cognitive deficits after mTBI. LOC was associated with cognitive deficits and slow recovery of cognitive function [25]. Among military personnel and civilian contractors, longer duration of LOC was associated with a greater decline in accuracy on the Automated Neuropsychological Assessment Metric (ANAM), which measures reaction time, learning, and memory, between baseline and post-injury tests [11]. Among veterans stratified into reduced or intact executive function subgroups, LOC was more prevalent in the reduced executive function subgroup [15]. Service members and veterans with LOC after combat-related mTBI reported memory problems [12,16]. Longer LOC (1–20 min) was associated with greater impairment rates in declarative memory and executive function tasks compared to mTBI with shorter LOC (<1 min) and control groups [21]. In an earlier study by the same team, longer LOC (1–20 min) was associated with deficits in event-based tasks of prospective memory (PM), defined as remembering the intention to perform a task, compared to the LOC < 1 min group but not the control group, whereas both LOC groups had impairments in time-based PM tasks [19]. Deficits in prospective memory may translate to a worse ability to perform daily tasks. This is consistent with a study reporting that LOC was associated with incomplete functional recovery, defined as the ability to perform activities of daily living, 1 and 3 months after mTBI compared to groups with altered mental state (AMS) and neither LOC nor AMS [23]. AMS is an interchangeable term for AOC, one of the criteria used to diagnose TBI. Altogether, these studies have reported a negative association between mTBI with LOC and cognitive performance as measured by executive function, reaction time, memory, and attention tests, which may have implications for daily functioning.

Interestingly, several studies have reported better cognitive performance associated with LOC following mTBI. For example, LOC duration was inversely correlated with forgetfulness and overall cognitive impairment in the Neurobehavioral Symptom Inventory (NSI) in male veterans [22]. However, no such correlation existed in female veterans, suggesting sex-specific effects on the relationship between LOC and these outcome variables [22]. In an attention switching task, the difference in reaction time to trials where the placement and direction of arrows on a screen are the same (congruent) or different (incongruent) is referred to as the congruency cost [26]. In this study, mTBI with LOC was associated with lower congruency cost in the attention switching task than mTBI without LOC, which indicated better cognitive performance as less time was needed to complete more difficult tasks [26]. Additionally, LOC was associated with improved visual working memory in students who had sustained mTBI resulting from sports or other causes [24]. One possibility presented by the authors is that LOC may have prompted patients to seek medical care; in contrast, patients not experiencing LOC may have returned to play before recovering fully and thus risked re-injury [24]. These studies indicate that the relationship between LOC and cognitive performance can be inconsistent, and more research needs to be done to reconcile these inconsistencies.

### 3.2. Psychiatric Disorders

LOC after mTBI is associated with the development of psychiatric disorders, including post-traumatic stress disorder (PTSD) and major depressive disorder (MDD). One study reported that LOC was associated with MDD and emotional lability, a condition characterized by exaggerated and rapidly fluctuating emotions [25]. Among patients stratified by age, sex, and LOC status, LOC was associated with development of anxiety, suicidal ideation, and MDD within a 180-day period after admission for mTBI [27]. Importantly, all age and sex subgroups with LOC had higher odds of developing MDD [27]. LOC was associated with greater development of MDD compared to AOC/AMS groups and other (non-TBI) injury groups in soldiers studied several months after deployment [10,12]. LOC after mTBI was associated with greater self-reported MDD and somatic symptom disorder (SSD) symptoms compared to the mTBI without LOC group and the non-TBI group in wounded service members [29]. Previous studies suggest that mTBI with LOC is associated with increased development of MDD and other psychiatric disorders. 

In addition to MDD, LOC after mTBI is associated with the development of PTSD. In soldiers studied several months after returning from deployment, LOC was associated with greater development of PTSD compared to the AOC/AMS groups and other (non-TBI) injury groups [10,12]. In service members sustaining mTBI after blast injury, LOC was associated with the development of PCS and PTSD after deployment [13]. LOC following blast-related mTBI was associated with acute stress reactions (ASRs), including dissociation, re-experiencing, avoidance, and hyperarousal in veterans [16]. These symptoms resemble those of PTSD, but they have a much more transient time course, typically resolving within a few days after a traumatic incident [16]. Similarly, LOC was associated with avoidance symptomology of PTSD, although not re-experiencing or hyperarousal, and lower psychological quality of life in veterans [18]. In contrast, LOC was associated with higher re-experiencing/intrusion cluster scores compared to head injury without LOC and no head injury groups at 8 months, and elevated PTSD symptom scores at both 10 days and 8 months after a motor vehicle accident [14]. In a longitudinal study following a trajectory of PTSD symptoms over a year after mTBI, LOC was associated with a chronic trajectory marked by consistently high PTSD symptom scores throughout the study [28]. Overall, these clinical studies provide evidence that mTBI with LOC may increase development of psychiatric disorders, including PTSD, compared to mTBI without LOC.

The relationship between LOC and PTSD is controversial, as unconsciousness may theoretically protect against trauma by preventing full awareness and processing of the traumatic event, as discussed in [29]. Interestingly, this study also reported that mTBI with LOC was associated with greater PTSD symptom self-reporting compared to the non-TBI group, but not compared to the mTBI without LOC group, which was unexpected [29]. In contrast, LOC was associated with more PTSD symptoms compared to the head injury groups and no injury groups at 10 days and 8 months after a motor vehicle accident, with elevated re-experiencing/intrusion cluster scores at 8 months post-injury [14]. Interestingly, these patterns emerged despite equal levels of fear and threat perception among groups during the accident [14]. Importantly, these findings remained the same when the data was re-analyzed with the omission of PTSD-related amnesia [14]. The authors suggest several explanations for these findings, such as impaired fear extinction due to psychological disturbances or neuronal damage; cognitive deficits which may impair ability to cope with and process the situation; physical symptoms which may serve as reminders of the event; or the traumatic memory being preserved, as the traumatic memories may be more pronounced with the absence of memories immediately following the incident [14]. Much of the literature on the relationship between LOC, PTSD, and memory is focused on more severe TBI; therefore, more research on these relationships in mTBI patients is warranted.

### 3.3. Physical Symptoms

LOC after mTBI is associated with various physical symptoms, including headache, dizziness, and balance problems. The duration of LOC in women, but not men, was positively correlated with loss of balance, poor coordination, fatigue, and overall vestibular impairment score on the NSI in a study comparing male and female veterans [22]. Additionally, LOC was associated with balance problems, fatigue, and headache; interestingly, every patient with LOC in this study reported headache [25]. In military personnel, LOC was associated with greater levels of plasma interleukin (IL)-6, a pro-inflammatory cytokine, and greater self-reported pain levels [20]. In soldiers recently returning from deployment, mTBI with LOC was associated with headache, balance problems, and musculoskeletal pain [12] or headache alone [10] compared to other injuries, with no association between AOC/AMS and physical symptoms [10,12]. Blast-related mTBI with LOC was associated with hearing loss and with difficulty sleeping that persisted at a follow-up visit 48-72 h later [16]. LOC after mTBI was associated with more self-reported somatic symptoms compared to mTBI without LOC and non-TBI groups in wounded service members [29]. Therefore, the presence and duration of LOC following mTBI increased the risk of developing adverse physical symptoms.

Several studies have demonstrated that psychological factors may underlie adverse health outcomes in mTBI patients. For example, adjustment for ASRs nullified the initial associations of LOC with ringing in ears and balance problems after blast-related mTBI in military personnel, and the remaining associations were weakened [16]. In soldiers surveyed several months after returning from deployment, mTBI with LOC was initially associated with almost all health-related outcomes and post-concussive symptoms measured when compared to other injuries [10,12]. However, few associations remained significant when adjusted for PTSD, MDD, and other factors [10,12]. Additionally, the remaining LOC-associated symptoms were more strongly associated with PTSD and MDD than with LOC [10,12]. This confounding effect may be explained by an overlap between adverse symptoms commonly reported after mTBI and those associated with PTSD and MDD, such as sleeping problems [10,12,18]. Taken together, these studies demonstrate that psychological factors partially influence poor physical health outcomes associated with LOC after mTBI.

### 3.4. Brain Abnormalities

Variations in LOC after mTBI are associated with structural brain abnormalities which may contribute to functional deficits such as cognitive, psychiatric, and physical symptoms. Magnetic resonance imaging (MRI) demonstrated that LOC was associated with greater thickness of the left and right rostral anterior cingulate cortices (rACC), which itself was predictive of a chronic PTSD somatology trajectory marked by high PTSD symptom scores with minimal improvement [28]. LOC was also associated with a chronic PTSD trajectory, indicating direct a relationship between brain abnormalities, PTSD symptomology, and LOC [28]. A variation of the standard MRI technique is diffusion tensor imaging (DTI), which uses the movement of water molecules to measure white matter integrity. Fractional anisotropy (FA), a measure of directional movement along the axon, is proportional to white matter integrity, while radial diffusivity (RD), which measures diffusion out of the axon, is inversely related to white matter integrity; in other words, low FA and high RD are indicative of impaired white matter integrity. A DTI study found that lower FA in the internal capsule was associated with greater PTSD symptoms in patients with LOC following blast-related mTBI [17]. Another DTI study revealed that LOC was associated with increased RD in the ventral prefrontal white matter compared to AOC and control groups [15]. This RD was increased in patients with reduced executive functioning, who were significantly more likely to have LOC; therefore, loss of white matter integrity in the ventral prefrontal area may explain the deficits in executive functioning observed in patients with LOC [15]. In summary, associations between LOC after mTBI and outcomes discussed previously have underlying neural correlates, which are themselves associated with LOC. While much of the literature has reported abnormalities in brain function and structure in patients with mTBI, only a handful of papers discuss a direct interaction between brain abnormalities, LOC status, and symptoms; more research on these interactions is warranted.

### 3.5. Conclusion on Clinical mTBI and LOC

In patients with mTBI, LOC can be a useful predictor of symptom development and severity. LOC has been associated with adverse effects such as deficits in cognition, learning, and memory; psychiatric disturbances such as PTSD and MDD; physical symptoms such as headache; and brain abnormalities, particularly in white matter areas. As such, one can predict that mTBI patients with LOC are more likely to develop adverse effects. Clinicians should triage mTBI patients with LOC and monitor them to prevent worsening symptoms, particularly in the context of return-to-play decisions in athletes or return to duty in military personnel. Additionally, literature on brain abnormalities has revealed associations with adverse outcomes. In particular, DTI analysis of white matter is a sensitive neuroimaging modality that is gaining popularity among clinicians and clinical researchers. LOC following mTBI may be a useful indication for this advanced imaging technique, allowing for more accurate detection of mTBI-related abnormalities that traditional techniques may miss.

## 4. Righting Reflex in Preclinical TBI Studies

**Figure 1:**
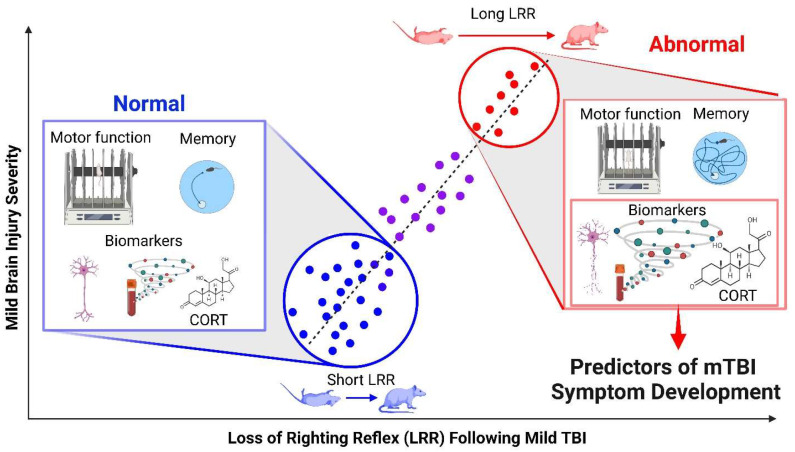
Individual variability in LRR and outcome measures after rodent TBI, which can be translated to human mTBI. Data is hypothetical. Increases in loss of righting reflex (LRR) are directly correlated with increases in symptom severity. Data points on the trend line represent individual rodents, with less and more symptomatic rodents in blue and red, respectively. Symptom severity in these rodents is graphically portrayed in the insets in the form of rotarod performance (motor impairments), Morris water maze performance (cognitive and memory deficits), neuronal degeneration and blood-based biomarkers (pathology), and corticosterone (CORT) levels (physiology). Image created with BioRender.com.

This section examines LRR as a predictor of outcomes after experimental TBI. The studies contained in this section are summarized in Table 2, and a graphic summary is provided in Figure 1.

### 4.1. Injury Parameters and Variability

Several preclinical studies have shown associations between increased injury parameter level and LRR. For instance, increasing the impact energy in a Closed-Head Impact Model of Engineered Rotational Acceleration (CHIMERA) injury produced correspondingly higher LRR in mice [40]. In this study, LRR was positively correlated with acceleration, velocity, and displacement of the head after impact [40]. Similarly, LRR was positively correlated with greater piston velocity in mice receiving a modified CHIMERA injury [47]. Moreover, in rats receiving a controlled cortical impact (CCI) injury, LRR was correlated with a greater depth of tissue deformation [30]. In rats receiving a lateral fluid percussion (LFP) injury, LRR was positively correlated with injury pressure [42]. Taken together, adjusting the level of injury parameters leads to variation in LRR, providing evidence that LRR is related to injury severity.

Previous preclinical studies demonstrated that increases in specific pre-set injury parameters can predictably increase LRR. Even in a TBI intended to be identical across animals, however, it is worth noting that the injury experienced by the rodent may be variable as a result of unintended experimental variation. A weight drop model, for example, has many components that can lead to unintended variabilities. In particular, slight differences in the angle of a protective helmet, commonly used in closed-head impact models to prevent skull fracture [55], may have led to appreciable differences in head acceleration [34]. Head acceleration can influence LRR directly [38,40] or through a mutual interaction with traumatic axonal injury (TAI), defined as the shearing of axons resulting from head acceleration during TBI [34]. In this study, TAI was quantified by the number of axonal swellings and retraction balls in the corpus callosum [34]. Furthermore, TAI is considered a potential mechanism leading to traumatic unconsciousness [56]. The synthesis of these studies represents a pathway by which minute experimental differences can contribute to significant variability in injury experience and influence LRR.

Inherent variations among individuals may also lead to heterogeneity in LRR in animal studies even though the animals were subjected to injuries with identical parameters. For example, mice receiving the same closed-head impact were separated into groups based on LRR and apnea, and these two groups had significant differences in head acceleration, behavior, and pathology [38]. Additionally, LRR was directly correlated with higher maximum vertical ipsilateral g-force of the head in high-LRR mice [38]. The authors proposed that individual variability between mice, such as small differences in head weight, shape, and volume, would explain variation in head acceleration that may in turn lead to more variability in LRR and contribute to group differences in behavioral deficits and pathology [38]. In summary, LRR can be correlated with pre-determined escalation of injury parameters, but even with the identical injury paradigms, a certain degree of variability in LRR is inevitable due to individual variations.

### 4.2. Motor and Sensorimotor Dysfunction

Preclinical studies have found that LRR is correlated with motor impairments in rodents. One preclinical test of motor function is the rotarod test, where a rodent must walk on a rotating rod at an accelerating speed, and latency to fall is a measure of motor coordination. The rotarod test is more sensitive at detecting motor changes in TBI vs. sham rodents compared to other commonly used motor tests such as the balance beam and beam walk tests [57,58]. A study utilizing a closed-head TBI model in mice showed that high-LRR mice had significantly lower rotarod latency than low-LRR mice on days 1 and 3 post-injury, with these deficits resolving by day 7 [38]. Similarly, LRR was correlated with worse rotarod performance 1 day after a closed-head injury in mice [45]. LRR was also correlated with worse rotarod performance at 1 h, 24 h, and 3 months after a single or repetitive (4 hourly impacts) mild projectile concussive impact (PCI) injury in rats [41]. Rodents with higher LRR performed worse on the rotarod test, a gold standard measure of motor coordination in preclinical literature. These motor deficits are consistent with balance problems and loss of coordination associated with LOC after mTBI.

In addition to motor deficits, LRR was also associated with worse performance on sensorimotor tests. LRR was positively correlated with impairments in gait at 1 h and 3 months after PCI injury as measured by CatWalk™ gait analysis in rats [41]. LRR was also correlated with a higher score on the Revised Neurobehavioral Severity Scale (NSS-R), a ten-item battery of sensorimotor tests in which a higher score indicates greater impairment, at 3 months after PCI injury in rats [41,59]. Another study found that LRR was correlated with higher Neurological Severity Score (NSS), a similar battery of sensorimotor tests in which a higher score indicates greater impairment, within 24 h after an LFP injury in rats [42]. Similarly, LRR was correlated with higher NSS 1 h after a moderate-severe CHIMERA injury in mice [44]. In summary, LRR in rodents is associated with impairments in sensorimotor function, consistent with neurological deficits and poor performance on neurobehavioral inventories associated with LOC after mTBI.

### 4.3. Cognitive and Memory Deficits

LRR is associated with contradictory effects on performance on tasks of attention. In rats experiencing weekly CHIMERA injuries, LRR was predictive of worse performance on most facets of the 5-choice serial reaction time task (5CSRT), which requires rodents to recall which of five holes was illuminated and nose poke it to receive a food reward, and on the delay discounting task (DDT) which requires rodents to choose between receiving a smaller food reward immediately or a larger food reward after a delay [51]. Another task of attention, the attentional set-shifting task (AST), utilizes two pots which can be discerned by smell and digging media, and the rodent must learn which cue (one of two smells, or one of two media) is associated with a reward buried in the pot, and cues are changed or introduced throughout the paradigm. Specifically, in rats undergoing 21 days of maternal separation (MS) or controls followed by mild CCI or sham on day 21, LRR was negatively correlated with the number of trials to criterion for intra-dimensional (ID) shift in which entirely new odor and digging media cues are introduced, such that rats with higher LRR learned faster, when AST was assessed on days 35-40 [46]. This is not entirely surprising, as contradictory cognitive effects were also observed in mTBI with LOC. LRR has mixed correlations with performance on attention tasks, consistent with improved performance on an attention switching task in mTBI with LOC.

Additionally, LRR is associated with mixed effects in tests of learning and memory. LRR had mixed associations with performance on object memory and recognition tasks. The novel object recognition test has rodents interact with two identical objects, one of which is later replaced with a new object, and time spent interacting with the old and new objects is compared; a rodent with intact memory would interact more with a new object. A study of mice receiving a moderate LFP injury showed that LRR was correlated with worse NOR performance on days 9 and 16 after injury [39]. A study of rats receiving midline fluid percussion (MFP) injury, in contrast, showed no correlation between LRR and the NOR, the novel object location (NOL) test, in which one of two identical objects is moved to a new location, and the temporal order object recognition (TOR) test, in which objects the rodent interacted with more and less recently are tested together, 3 and 6 months post-injury [53]. LRR was consistently correlated with impairments in spatial memory tests where a rodent must learn the location of an escape platform in a pool of water (Morris water maze), a shock zone segment of a rotating circular arena (active place avoidance [APA]), and a dark escape compartment accessed by only one of the many holes on the perimeter of a circular arena (Barnes maze). After a weight drop injury, LRR was correlated with impaired Morris water maze retention in moderately (LRR ≤ 12 min) and severely injured (LRR > 12 min) rats, and impaired learning in severely injured rats 1 week post-injury [32]. After a moderate LFP injury in rats, LRR was correlated with greater time taken to complete the Barnes maze [33]. Similarly, LRR was correlated with impairments on the Barnes maze probe trial 19 days after a CHIMERA injury in mice [44]. In separate cohorts of mice undergoing the APA test 7 days or 1 month after a closed-head injury, high-LRR mice had impaired learning and retention of the shock zone location, while low-LRR mice only had impaired retention of the shock zone location [38]. LRR has been associated with impairments on numerous tests of attention, learning, and memory, consistent with learning and memory deficits in mTBI with LOC, although there have been inconsistent results in both clinical and preclinical literature.

### 4.4. Central and Peripheral Pathology

LRR after experimental TBI is correlated with elevation of a number of central and peripheral biomarkers. Serum cytokines and microRNAs were released 1 and 24 h after PCI injury in rats, and this release was positively correlated with LRR at these time points [41]. Another study showed that LRR was correlated to higher plasma cell-free DNA, a clinical marker of poor TBI prognosis, 2 h after repeated blast injuries in mice [36]. LRR was positively correlated with hippocampal and cortical levels of glial fibrillary acidic protein (GFAP), a marker of astrocytes, 24 h after PCI injury in rats [41]. LRR was correlated with greater phosphorylated neurofilament heavy (pNfH) and GFAP levels in both the cerebrospinal fluid (CSF) and serum 24 h after a weight drop injury in rats [37]. In mice receiving a single impact with a controlled electromagnetic impactor, LRR was correlated with higher plasma levels of neurofilament light (NfL), another clinically relevant TBI biomarker, a week after the injury [54]. Furthermore, LRR in mice was correlated to serum levels of neuron-specific enolase (NSE), a biomarker for TBI severity, 24 h after a weight drop injury [35]. In rats undergoing 21 days of MS with a CCI injury on day 21, LRR was correlated with levels of IL-1β, a pro-inflammatory cytokine, in the ipsilateral hippocampus on day 42; however, caution should be taken when interpreting these results, as this correlational analysis examined stressed and unstressed rats jointly, and as such, stress may be a confounding variable [46]. The release of peripheral biomarkers may be explained by increased blood–brain barrier permeability; in mice receiving blast injuries, LRR was correlated with the degree of Evans-Blue staining, an indicator of blood–brain barrier breach, on the surface of the cerebral cortex 4 h after the injury [52]. Interestingly, 1 week after an LFP injury in rats, LRR was not correlated with plasma fluorescein isothiocyanate–labeled dextran (FD4), a marker of intestinal barrier integrity, or lipopolysaccharide (LPS), a bacterial endotoxin, suggesting that LRR has no correlation with permeability in the intestines, in contrast to the blood–brain barrier [48]. A summary of the literature indicates that LRR after experimental TBI is correlated with elevated levels of a number of peripheral and central biomarkers. Many of these biomarkers, specifically GFAP, NfH, NfL, microRNAs, and NSE, are currently being studied in humans, although their clinical utility is variable [60]. Examination of these biomarkers in rodents may shed light on targets for human treatment.

Many studies have found associations between LRR and neuronal damage. A study comparing rats receiving either an LFP injury or a weight drop impact acceleration followed by hypoxia (WDIA + H) injury found that neuronal degeneration, as indicated by Fluoro-Jade B (FJB)-positive cell count throughout the brain, was correlated with LRR in WDIA+H rats [9]. Further, after a moderate pendulum strike injury in rats, LRR was associated with greater silver staining intensity, a measure of axonal degeneration in the brain, at 48 h but not 1 week after the injury [31]. The authors proposed that this relationship did not persist after 1 week because of the accumulation of neurodegeneration [31]. In a PCI study in rats, LRR was correlated with neuronal degeneration in the ipsilateral cortex as measured by FJB staining 24 h after injury [41]. In rats receiving a weight drop injury, LRR was correlated with traumatic axonal injury (TAI) in the corpus callosum 24 h after injury [34]. After a closed-head injury, high-LRR mice had greater axonal loss and demyelination in numerous white matter regions than low-LRR mice and shams 1 month after a closed-head injury [38]. The authors noted that demyelination in the fimbria of the hippocampus, observed only in high-LRR mice, was previously associated with poor learning in the APA task [38]. One reason for this difference in white matter pathology may be due to greater vertical g-force experienced in high-LRR mice, as well as greater compression of the skull as indicated by hemorrhaging [38]. This parallels the changes observed in clinical studies, particularly for white matter degradation related to cognitive deficits in rodents with higher LRR and mTBI patients with LOC.

### 4.5. Physiological Abnormalities

In addition to the changes mentioned above, LRR in rodents has also been associated with several physiological changes after TBI. For example, LRR was correlated with elevated serum corticosterone (CORT), the rodent analogue to the human stress hormone cortisol, 1 h after PCI injury in rats [41]. LRR was correlated with levels of plasma CORT on day 42 in rats receiving 21 days of maternal separation followed by a CCI on day 21; however, caution should be taken when interpreting these results, as this correlation analysis examined stressed and unstressed rats jointly, and as such, stress may be a confounding variable [46]. Additionally, LRR was correlated with elevated CORT in the contralateral hippocampus 3 days after an LFP injury in rats; surprisingly, the correlation with CORT in the ipsilateral hippocampus was not significant [49]. In the same study, LRR was also correlated with a longer duration of seizures immediately after the injury, and LRR was higher in rats with cyanosis and acute mortality [49]. In a severe LFP model in rats, LRR was inversely correlated with mean post-impact arterial O_2_ saturation within 5 min of injury and the number of seizures within 1 week of injury [43]. Interestingly, a study of rats receiving an MFP injury showed that LRR was not correlated with cerebral arterial dilatation, cerebral blood flow, or cerebral blood volume 6 months after injury, indicating inconsistent correlations with arterial and circulatory function [53]. A closed-head injury study showed that mice with prolonged apnea had higher LRR and mortality, whereas mice with no apnea had low LRR and no fatalities [38]. In a recent study of repetitive weight drop injury for five consecutive days in mice, LRR was correlated with greater percent body weight loss compared to baseline on injury days 2 to 5 [50]. These studies provide evidence that LRR is correlated with various physiological abnormalities, including high brain and blood CORT levels and weight loss, with indeterminate correlations with respiratory/circulatory function and seizure duration and time. These preclinical findings are consistent with various physical symptoms reported in clinical studies in mTBI with LOC.

### 4.6. Conclusion for Preclinical TBI and LRR

A growing body of preclinical studies suggests that variations in LRR are associated with several adverse effects such as motor and sensorimotor deficits, cognitive and memory deficits, pathology in the brain and periphery, and physiological abnormalities. As such, LRR variations may predict TBI-related symptoms in rodents, similar to LOC in clinical TBI studies. In addition, by investigating rodents with higher LRR, one can examine the biological mechanisms underlying the propensity for longer LRR and greater symptom development, which may provide insight into the pathophysiological consequences of LOC duration in mTBI as well as novel pharmaceutical target developments for related post-injury symptoms.

## 5. Discussion

### 5.1. Limitations

Although a majority of preclinical studies found differences in LRR between sham and various injured groups (such as [38,40,41,46]), it is not always the case. For example, among mice that received weight drop brain injuries, LRR was not different among injured mice with and without cortical spreading depolarization (CSD), a wave of sustained depolarization that adversely affects brain function [61]. Similarly, mice lacking the Sarm1 gene, which mediates axonal degeneration, and wild-type mice receiving weight drop or sham injuries had no differences in LRR within each injury condition (e.g., TBI WT = TBI Sarm1 KO), although the overall TBI and sham groups did have different LRR [62]. Additionally, LRR-matched rats receiving either LFP or WDIA+H injuries had stark differences in pathology and behavioral deficits such that LFP rats showed significantly worse impairments, suggesting that TBI paradigm may dictate differences in symptom presentation [9]. Interestingly, in the same study, LRR was still correlated with neuronal degeneration in the WDIA+H rats, despite this group having lower pathology scores compared to the LFP group [9]. This indicates that variations in LRR may be predictive of individual symptom development even if they do not predict group differences. Thus, it is recommended that preclinical researchers examine LRR not just as a direct measure of injury severity but as a predictor of outcomes after experimental TBI.

A methodological factor to consider in TBI studies with LRR is the effects of single vs. multiple injuries. Mountney et al. [41] administered one or four PCI injuries to rats, one injury per hour, and LRR was higher in the second through fourth impacts than in the first [41]. In other studies, however, LRR was decreased over additional impacts. One study with a weight drop model where mice received injuries once a day for 30 days, injured animals showed greater LRR for the first 13 impacts, but not with subsequent impacts [63]. The authors attributed this phenomenon to central nervous system adaptation to subsequent impacts [63]. In another weight drop model in mice receiving 0, 1, 10, or 15 impacts over 23 days on a schedule of three impact or sham days followed by two rest days, mice in the 15-impact group had prolonged LRR on impacts 1–6 compared to sham controls, but there were no differences between the groups after impacts 7 to 15 [64]. The authors suggest that the mice with more impacts and fewer days off did not have time to adequately heal. The peak and then fall seen in the Hiskens et al. [64] study may be attributed to accumulation of neuronal damage over time, and that the neuronal adaptation only begins past a certain threshold of damage. Interestingly, more time between impacts could allow for either healing and adaptation or more accumulation of damage. This would be worth investigating and could be approached with a time course analysis of brain tissue over several injury days.

Anesthesia is used to induce LRR in rodents [65], at doses comparable to those used to induce human LOC [66]. Because animals are usually anesthetized prior to TBI, anesthesia can be a major confounding variable in the analysis of LRR following experimental TBI. In mice receiving inhalant halothane, intravenous propofol, or injectable chloral hydrate administered alone or before closed-head TBI, chloral hydrate induced an LRR of up to 90 min, which obscured injury effects and delayed behavioral testing [67]. Thus, the type of anesthetic can have a profound effect on LRR. Using inhalant anesthesia with a minimum exposure would be preferable over injectable anesthetics as it produces much more transient LRR, as well as being easier to implement. The duration of anesthesia is another point to consider. In groups of rats receiving either rotational acceleration or blast-related injury, both sham and injured rats in the rotational acceleration group showed greater LRR, which was attributed solely to a longer anesthetic exposure compared to the blast-related injury group [68]. Therefore, the type and duration of anesthesia can influence LRR, and this should be considered when conducting TBI experiments.

### 5.2. Future Directions

Most studies in this review utilize correlational analysis between LRR and outcome variables. While this is a valuable approach because it demonstrates predictive value, it has weaknesses in that it does not demonstrate a causative relationship. To address this, an underutilized approach is to stratify rodents into subgroups based on LRR. A study by Grin’kina et al. [38] stratified mice into groups based on apnea and LRR after closed-head injury, and these two subgroups had differences in spatial learning, white matter pathology, and motor deficits [38]. Similarly, in a study by To et al. [47] which stratified mice by LRR after modified CHIMERA hits of varying velocity, the high-LRR group had more intense neuronal degeneration and microgliosis in white matter tracts [47]. In addition, the high-LRR group had more profound and widespread neuronal damage as measured by DTI and neurite orientation dispersion and density imaging (NODDI), another MRI technique which measures neuronal density and orientation based on the diffusion patterns of water molecules [47]. Stratifying rodents into groups based on LRR allows for a direct comparison between high and low LRR groups of rodents. This approach may also be more clinically relevant as the duration of LOC following mTBI can rarely be measured precisely, unlike LRR after experimental TBI in rodents. Thus, LOC is generally treated as a categorical variable rather than a continuous one in most clinical mTBI studies. Therefore, differences between high-LRR and low-LRR groups of rodents may better approximate the differences between mTBI patients with different durations of LOC (i.e., <1 min, 1–20 min, and 20–30 min) or between mTBI with or without LOC. Subgroup analysis also reflects the reality that only a subset of the mTBI population develops symptoms, unlike in rodents where injuries are engineered to achieve a particular level of damage.

The current review highlights the relationship between LOC and adverse outcomes after clinical mTBI and the significance of variations in LRR following TBI in rodents and its functional significance in predicting vulnerability to developing TBI-related symptoms in rodents. Translation between preclinical and clinical studies of TBI using clinically relevant measures such as LRR and LOC is promising in advancing prognostic and therapeutic strategies to treat debilitating head trauma in military service members and in the general population. LRR is an appropriate proxy for LOC, with both being associated with similar outcome measures such as motor disturbances, white matter pathology, and cognitive deficits. This allows preclinical studies using LRR to be approximated to human studies using LOC, and findings may be translational. Because of this, examining rodents with longer LRR can be used to find mechanistic targets underlying adverse outcomes for TBI, and this information can be applied in humans as well to inform therapeutic targets for the treatment of TBI, as there are currently no FDA-approved pharmaceutical treatments for TBI [69,70]. However, this approach has potential as there are many mutual biomarkers between humans and rodents, and investigation into these mutual biomarkers and their mechanisms in TBI may lead to the development of pharmaceutical treatments.

To improve prognosis of mTBI and prevent adverse symptom development in a subgroup of patients, other available resources should be considered as well. Blood-based biomarkers and advanced MRI techniques such as DTI and NODDI can help clinicians identify vulnerable patients and intervene earlier in the injury process. Blood-based biomarkers may be a valuable resource, as several biomarkers examined in human mTBI [60] were found to be correlated with LRR in rodents, particularly GFAP [37], NfL [54], NfH [37], NSE [35], and microRNAs [41]. Serum ubiquitin C-terminal Hydrolase-1 (UCH-L1) is able to distinguish mTBI patients with a GCS of 15 from non-injured controls and from trauma controls with non-head injuries [71]. Additionally, serum GFAP and UCH-L1 have been FDA-approved for ruling out the need for a head CT in patients with mild and moderate TBI, and these markers performed similarly well in the mild TBI cohort (GCS 14-15) and the combined mild and moderate cohort (GCS 9-15) [72]. By taking a multimodal approach of integrating precise LOC information, FDA-approved blood-based biomarkers, physiological information, and cutting-edge brain imaging, clinicians can develop an evidence-based prediction strategy of mTBI symptoms in vulnerable individuals. Overall, LOC and LRR data can serve as an important measure of TBI severity and may help identify individuals vulnerable to adverse sequelae of head injury. Paired with advanced brain imaging and blood-based biomarkers, LOC and LRR data have important implications in advancing mTBI research and clinical practice in military and civilian populations.

## Figures and Tables

**Table 1:** A summary of clinical studies. mTBI = mild traumatic brain injury; LOC = loss of consciousness; AMS = altered mental status/altered mental state; MDD = major depressive disorder; PTSD = post-traumatic stress disorder; ANAM = Automated Neuropsychological Assessment Metric; AOC = alteration of consciousness; EMED = Expeditionary Medical Encounter Database; PCS = post-concussive/post-concussion syndrome; ASR = acute stress reaction; FA= fractional anisotropy; QoL = quality of life; CLSA = Canadian Longitudinal Study on Aging; PM = prospective memory; IL-6 = interleukin 6; PTA = post-traumatic amnesia; NSI = neurobehavioral symptom inventory; HeadSMART = Head injury Serum Markers for Assessing Response to Trauma; AST = attention switching task; rACC = rostral anterior cingulate cortex; MS-TBI = moderate-to-severe TBI; SSD = somatic symptom disorder.

Author, Year	Patient Population	Nature/Cause of Injury	Timing of Assessment	Groups	Outcomes
Hoge et al., 2008 [10]	Soldiers returning frrom Iraq	Blast or explosion, bullet, fragment or shrapnel, fall, vehicle accident, other	3–4 months after deployment	mTBI with LOC mTBI with AMS Other injury No injury	LOC associated with headache, MDD, PTSD
Luethcke et al., 2011 [11]	Military personnel and civilian contractors in Iraq	Blast injury, non-blast injury (blunt object, sport/recreation, falls, motor vehicle accident)	Within 72 h of injury	Blast mTBI Non-blast mTBI No LOC LOC >1 min LOC 1–20 min LOC <20 min	LOC duration correlated with greater decline in ANAM accuracy scores between baseline and post-injury tests
Wilk et al., 2012 [12]	Soldiers returning from Afghanistan and Iraq	Blast/explosion, bullet, fragment/shrapnel, fall, vehicle crash, or other	4–6 months after deployment	Single AOC Single LOC Multiple AOC Multiple (1+) LOC Other injuries No injury	LOC associated with MDD, PTSD, headache, memory problems, balance problems, muskulosekeltal pain
Eskridge et al., 2013 [13]	Retrospective study of male service members in Iraq from the EMED	Blast-related injury	mTBI diagnosed within 48 h of injury; variable follow-up	mTBI with LOC mTBI without LOC	LOC associated with PTSD and PCS
Roitman et al., 2013 [14]	Motor vehicle accident survivors	Motor vehicle accident	Admission average of 1.5 h after the accident; PTSD evaluation 10 days and 8 months later	LOC Head injury No head injury	LOC associated with elevated PTSD scores at 10 days and 8 months vs. head injury and no head injury groups; elevated PTSD prevalence and re-experiencing/intrusion cluster scores 8 months post-injury
Sorg et al., 2014 [15]	Afghanistan and Iraq war veterans	Blunt or blast injury	Variable	mTBI with LOC mTBI with AOC Controls	LOC associated with reduced executive functioning, reduced ventral prefrontal white matter integrity
Norris et al., 2014 [16]	Military personnel in Afghanistan	Blast-related injury	mTBI diagnosis within 72 h of injury; follow-up 48–72 h later	mTBI with LOC mTBI without LOC	LOC associated with ASRs, memory problems, hearing loss, difficulty sleeping, increased symptom reporting
Hayes et al., 2015 [17]	Afghanistan and Iraq war veterans	Blast-related injury	Variable	mTBI with LOC mTBI without LOC Controls	Lower internal capsule FA associated with greater PTSD symptom severity in LOC group
Sofko et al., 2016 [18]	Afghanistan and Iraq war veterans	Fragments, bullets, vehicular accidents, falls or blasts	Shortly following intake for PTSD treatment	mTBI with LOC mTBI without LOC	LOC associated with avoidance, lower psychological QoL, and more post-concussive symptoms
Bedard et al., 2018 [19]	mTBI patients from CLSA cohort	Not specified	1 year or more after mTBI	LOC <1 min LOC 1–20 min Controls	LOC 1–20 min associated with worse performance on event-based PM tasks compared to LOC < 1 min, but not compared to controls; both LOC groups had impairments in time-based PM tasks
Kanefsky et al., 2019 [20]	Active duty military personnel recruited from sleep study cohort	Not specified	3–18 months after returning from deployment	mTBI with LOC mTBI without LOC Controls	LOC associated with higher pain self-reporting and higher levels of plasma IL-6
Bedard et al., 2020 [21]	mTBI patients from CLSA cohort	Not specified	1 year or more after mTBI	LOC < 1 min LOC 1–20 min Controls	LOC 1–20 min associated with higher impairment rates in declarative memory and executive functioning tasks
Gray et al., 2020 [22]	Retrospective study of veterans from Polytrauma Network Site	Blasts, motor vehicle accidents, falls, blunt trauma	Variable	Men or women mTBI with LOC mTBI with AOC mTBI with PTA	-LOC duration correlated with loss of balance, poor coordination, fatigue, worse vestibular score on NSI in women -LOC duration correlated with less forgetfulness and better cognitive score on NSI in men
Roy et al., 2020 [23]	mTBI patients from HeadSMART cohort	Blunt head trauma by pedestrian struck, motor vehicle collision, fall, assault, struck by or against and object, bicycle collision, other, intoxication by drugs or alcohol	Medically evaluated within 24 h of mTBI; functional recovery assessed 1, 3, 6 months after TBI	AMS only LOC only LOC and AMS Neither LOC nor AMS	LOC associated with incomplete functional recovery 1 and 3 months after injury
Arciniega et al., 2020 [24]	Undergraduate students with mTBI	Closed-head injury from non-sport causes or individual, high-impact, or team sports	Average of 4 years after injury	mTBI with LOC mTBI without LOC Controls	LOC associated with better visual working memory
Vanier et al., 2020 [25]	mTBI patients in litigation for brain injury	Motor vehicle accidents, fall, assault, other	Variable	mTBI with LOC mTBI without LOC	LOC associated with balance problems, MDD, fatigue, emotional lability, headache, cognitive deficits with slow recovery
Karlsen et al., 2021 [26]	mTBI patients in Trondheim mTBI follow-up study	Fall, violence, bicycle, sport motor vehicle accident, struck object, other	Approximately 2 weeks following mTBI	mTBI with LOC mTBI without LOC Community controls Trauma controls	LOC associated with lower congruence cost (better performance) on AST
Shahrestani et al., 2022 [27]	Retrospective cohort analysis of mTBI patients from Nationwide Readmission Database	Not specified	Followed until readmission within 180 days after primary admission	mTBI with LOC mTBI without LOC Male or female Age <26, 26–50, 51–75, >75 years old	LOC patients had higher rates of MDD in all groups, age- and sex-dependent increases in anxiety and suicidal ideation
Kosaraju et al., 2022 [28]	mTBI patients from trauma center study of serum biomarkers and PTSD	Interpersonal, motor vehicle accident, other	Enrolled at initial ED visit; PTSD symptom evaluation 1, 3, 6, 12 months after enrollment	mTBI with LOC mTBI without LOC	LOC associated with chronic PTSD profile, thickness in left and right rACC
Kim et al., 2023 [29]	mTBI or MS-TBI service members in Iraq and Afghanistan	Not specified	Initial intake within a few days of injury, initial assessment up to 72 h later, follow-ups 0–75 days (AP1) and 90–365 days (AP2) post-injury	mTBI with LOC mTBI without LOC MS-TBI Non-TBI	mTBI with LOC associated with: -higher MDD and SSD vs. mTBI without LOC -higher PTSD, MDD, and SSD vs. non-TBI

**Table 2:** A summary of preclinical studies. CCI = controlled cortical impact; LRR = loss of righting reflex; LFP = lateral fluid percussion; WDIA + H = weight drop impact acceleration + hypoxia; TAI = traumatic axonal injury; NSE = neuron-specific enolase; pNfH = phosphorylated neurofilament heavy; CSF = cerebrospinal fluid; GFAP = glial fibrillary acidic protein; NOR = novel object recognition; CHIMERA = Closed-Head Impact Model of Engineered Rotational Acceleration; PCI = projectile concussive impact; NSS-R = revised neurobehavioral severity scale; CORT = corticosterone; NSS = neurological severity score; ID = intradimensional shift; AST = attentional set-shifting task; IL-1β = interleukin 1 beta; FD4 = fluorescein isothiocyanate–labeled dextran; LPS = lipopolysaccharide; 5CSRT = 5-choice serial reaction time task; DDT = delay discounting task; MFP = midline fluid percussion; NOL = novel object location; TOR = temporal order object recognition; NfL = neurofilament light.

Author, Year	Species, Sex, Strain	Injury Model	Behavioral Outcomes	Biological Outcomes
Dixon et al., 1991 [30]	Rats, n.s., Sprague Dawley	CCI	-	LRR correlated with magnitude of tissue deformation (r = 0.78)
Morehead et al., 1994 [31]	Rats, male, Wistar	Pendulum strike	-	LRR correlated with silver stain pathology 48h (ρ = 0.9312) after injury or when 48h and 1 week groups were combined (ρ = 0.6845); not significant at 1 week alone (ρ = 0.4873, *p* > 0.1)
Schmidt et al., 2000 [32]	Rats, male, Sprague Dawley	Weight drop	LRR inversely correlated with learning (R^2^ = 0.7297) and retention (R^2^ = 0.6613) on Morris water maze 1 week post-injury	-
Hallam et al., 2004 [9]	Rats, male, Sprague Dawley	LFP, WDIA+H	-	LRR correlated with total neuronal degeneration in WDIA+H (r = 0.597)
Fedor et al., 2010 [33]	Rats, male, Sprague Dawley	LFP	LRR correlated with higher average time to complete Barnes maze (r = 0.656)	-
Li et al., 2011 [34]	Rats, male, Sprague Dawley	Weight drop	-	LRR correlated to TAI counts in corpus callosum (R^2^ = 0.545 or 0.549) 24 h post-injury
Goodman et al., 2013 [35]	Mice, male, C57/BL6	Weight drop, pre-treatment with water or ehanol	-	LRR correlated with serum levels of NSE (ρ = 0.65 in water animals, ρ = 0.6 in ethanol animals) 24 h post-injury
Wang et al., 2014 [36]	Mice, male, C57BL/6J	Repetitive (3×) blast	-	LRR correlated to cell-free DNA levels in plasma (r = 0.7) 2 h post-injury
Li et al., 2015 [37]	Rats, male, Sprague Dawley	Weight drop	-	LRR correlated with pNfH in CSF (R^2^ = 0.415) and serum (R^2^ = 0.204), GFAP in CSF (R^2^ = 0.427) and serum (R^2^ = 0.207)
Grin’kina et al., 2016 [38]	Mice, male, C57/BL6	Closed-head injury	High-LRR mice had impairments on rotarod test and active place avoidance learning and retention	High-LRR mice had lower axonal survival and more demyelination compared to low-LRR mice and sham; LRR correlated with maximum vertical ipsilateral g-force of head in high-LRR mice (ρ = 0.724)
Ouyang et al., 2017 [39]	Mice, male, C57BL/6	LFP	LRR correlated with worse NOR performance on post-injury days 9 (r = 0.778) and 16 (r = 0.769)	-
Namjoshi et al., 2017 [40]	Mice, male, C57BL/6	CHIMERA	-	LRR correlated with linear head displacement (ρ^2^ = 0.34), velocity (ρ^2^ = 0.3768) and acceleration (ρ^2^ = 0.2931), angular velocity (ρ^2^ = 0.3036) and acceleration (ρ^2^ = 0.2773)
Mountney et al., 2017 [41]	Rats, male, Sprague Dawley	Single and repetitive (4×) PCI	LRR correlated with higher NSS-R scores, poor rotarod performance, gait alterations	LRR correlated with higher serum levels of CORT, cytokines, microRNAs, higher brain GFAP, corpus callosum thinning
Smith et al., 2018 [42]	Rats, male, Wistar	LFP	LRR correlated with higher NSS (R^2^ = 0.47) within 24 h of injury	LRR correlated with pressure of impact (R^2^ = 0.28)
Andrade et al., 2019 [43]	Rats, male, Sprague Dawley	LFP	-	LRR inversely correlated with mean post-impact arterial O_2_ saturation (r = −0.74) and seizure number (r = −0.59)
Bashir et al., 2020 [44]	Mice, male and female, C57BL/6	CHIMERA	LRR correlated with higher NSS 1 h post-injury (ρ = 0.7702) and worse Barnes maze performance on day 19 (ρ = −0.4529)	-
Enam et al., 2020 [45]	Mice, male, C57BL/6N	Closed-head impact	LRR inversely correlated with day 1 rotarod performance (r = −0.53)	-
Lajud et al., 2021 [46]	Rats, male, Sprague Dawley	CCI	LRR correlated with fewer trials to criterion in ID shift of AST	RR correlated with elevated IL-1β expression in ipsilateral hippocampus (r = 0.458), plasma CORT levels (r = 0.391) 21 days post-injury
To et al., 2021 [47]	Mice, male, C57BL/6	Modified CHIMERA	-	LRR correlated with impact velocity (R^2^ = 0.55)
Mazarati et al., 2021 [48]	Rats, male, Sprague Dawley	LFP	-	LRR not correlated with plasma FD4 or LPS 1 week after injury
Komoltsev et al., 2021 [49]	Rats, male, Wistar	LFP	-	LRR correlated with longer duration of immediate seizures (r = 0.37 for right side righting, r = 0.35 for left side righting), CORT elevation in contralateral hippocampus on day 3 (r = 0.65)
Kahriman et al., 2022 [50]	Mice, male, C57BL6/J	Repetitive (5×) weight drop	-	LRR correlated with body weight loss on injury days 2–5 (r = −0.517, −0.651, −0.674, −0.748, respectively)
Vonder Haar et al., 2022 [51]	Rats, male, Long Evans	CHIMERA	LRR predictive of worse performance on 5CSRT and DDT	-
McNamara et al., 2022 [52]	Mice, male and female, C57BL/6J	Blast	-	LRR correlated with Evans-Blue staining in outer cerebral cortex 4 h post injury (τ = 0.508)
Griffiths et al., 2022 [53]	Rats, male, Sprague Dawley	MFP	LRR not correlated with NOR, NOL, TOR at 3 and 6 months post-injury	LRR not correlated with cerebral arterial dilation, blood flow, or blood volume at 6 months post-injury
Moro et al., 2023 [54]	Mice, male and female, C57BL/6J	Single or repetitive impacts with electro-magnetic impactor		LRR correlated with plasma NfL levels one week after a single impact injury (r = 0.67 or 0.75)

## Data Availability

Not applicable.
